# Multifunctional terahertz metamaterials device based on a dual-tunable structure incorporating graphene and vanadium oxide

**DOI:** 10.1039/d5na00278h

**Published:** 2025-05-12

**Authors:** Wenchao Zhao, Xiaowei Lv, Qianqian Xu, Zhengji Wen, Yuchuan Shao, Changlong Liu, Ning Dai

**Affiliations:** a School of Electronic Information, Huzhou College Huzhou 313000 China zhaowenchao2025@163.com; b Huzhou Key Laboratory of Urban Multidimensional Perception and Intelligent Computing Huzhou 313000 China; c Key Laboratory of Materials for High-Power Laser, Shanghai Institute of Optics and Fine Mechanics, Chinese Academy of Sciences Shanghai 201800 China wenzhengji@siom.ac.cn; d Hangzhou Institute for Advanced Study, University of Chinese Academy of Sciences Hangzhou 310024 China

## Abstract

This paper presents a multifunctional terahertz device based on a dual-tunable structure incorporating graphene and vanadium oxide (VO_2_). This device enables the switching between narrowband perfect absorption and ultra-broadband performance through the phase transition characteristics of VO_2_ and the adjustment of graphene Fermi level. Simulation results demonstrate that when VO_2_ is in its metallic state, the THz device exhibits ultra-broadband absorption, achieving a high absorption rate exceeding 0.9 within the frequency range of 2.9–7.67 THz. Conversely, when VO_2_ is in its insulating state, the THz device displays perfect absorption peaks at 2.8 and 8.41 THz. In the broadband mode, the absorption band can be broadened to an ultra-broadband range by adjusting the Fermi level of graphene. Furthermore, the structural parameters of terahertz devices, as well as the incident and polarization angles of electromagnetic waves, were investigated. The results demonstrated that the terahertz devices exhibit a certain degree of manufacturing tolerance, stability against variations in incident angles, and favorable polarization insensitivity. Overall, this design holds promising application prospects in fields such as terahertz absorption, refractive index sensing, and terahertz detection.

## Abstract 2

Here, a multifunctional terahertz device based on the dual tunable structure of graphene and VO_2_ combined structure is proposed.

## Graphical abstract

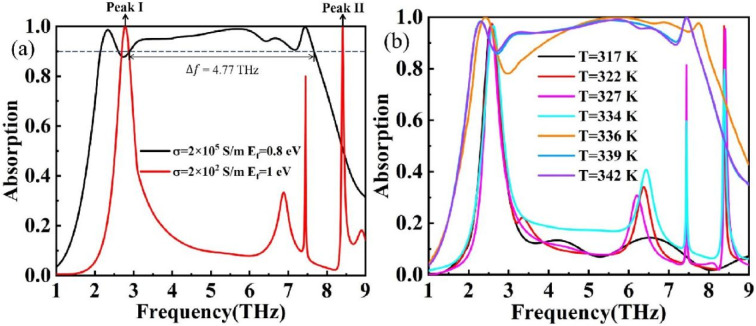

## 1. Introduction

Terahertz (THz) waves are broadly defined as electromagnetic waves with frequencies ranging from 0.1 to 10 THz, lying between the infrared and microwave regions of the spectrum. They occupy a transitional zone between macro-electronics and micro-photonics, possessing frequencies higher than those of microwaves but lower than infrared radiation, and their energy levels are situated between those of electrons and photons.^1–3^ Due to their unique characteristics of high transmission, high resolution, and low energy, terahertz waves have found increasingly widespread applications, becoming a research hotspot in recent years. Consequently, THz devices based on terahertz technology have also garnered significant research attention.^4–6^

Metamaterials, characterized by their regular arrangement and artificially controllable structure, enable the modulation of THz device absorption through the arrangement and combination of their constituent structures.^7^ In comparison to natural or traditional materials, metamaterial technology presents novel research avenues for the advancement of terahertz technology. Metamaterial absorbers, in particular, exhibit characteristics such as broad absorption bandwidths, multi-component structures, and reduced weight, which distinguish them from conventional absorbers. These attributes have led to the application of metamaterials in various fields including optoelectronic devices, wireless communications, and stealth fighter aircraft. Consequently, metamaterial technology has emerged as one of the key research foci.^8–10^ The first metamaterial THz device was proposed by Landy *et al.*, which proved the feasibility of metamaterials in the field of terahertz devices.^11^ As a result, numerous scholars have embarked on extensive research into THz devices utilizing metamaterials. In 2009, Bao *et al.* designed and fabricated a broadband microwave THz metamaterial device based on dendritic structural units, achieving high absorption within the frequency range of 9.79 GHz to 11.72 GHz.^12^

Graphene has emerged as one of the most popular research materials in the field of metamaterials in recent years, and terahertz devices incorporating graphene-based metamaterials have been extensively studied and reported.^13–15^ In 2004, the research group led by Geim and Novoselov discovered graphene under a microscope. This remarkable material boasts a thickness of merely 0.34 nm.^16^ Terahertz devices derived from graphene-based research have garnered widespread attention and emerged as one of the hotspots in recent research endeavors. Numerous reports have documented the development of graphene-based broadband or narrowband absorbers. Xie *et al.* proposed a multi-frequency narrowband perfect absorber utilizing a metamaterial structure patterned with a single layer of graphene, showcasing four absorption peaks with excellent performance in the infrared spectrum.^17^

VO_2_ exhibits remarkable optical properties at terahertz wavelengths, with its phase transition characteristics forming the basis for its applications in this spectral range. The phase transition temperature of VO_2_ is approximately 340 K, making it easily achievable under both experimental and practical conditions in ambient environments.^18–20^ During the insulator-to-metal phase transition, the conductivity of VO_2_ steadily increases, varying within the range of 2 × 10^2^ S m^−1^ to 2 × 10^5^ S m^−1^, accompanied by a transformation in its crystal structure from monoclinic to tetragonal. Compared to the separate structure of graphene, THz devices incorporating VO_2_ possess a broader tuning capability.^21^ The metal-to-insulator transition properties of vanadium dioxide can be used to achieve perfect absorption and asymmetric transmission (AT) of circularly polarized light in the near-infrared region through thermal control.^22^

In recent years, research on terahertz devices has extended beyond simple Fermi level tuning. Multilayer structures based on VO_2_ and graphene have garnered extensive investigation. Although slightly more complex in fabrication compared to three-layer graphene THz devices, advancements in manufacturing processes are poised to unlock the formidable potential of multilayer THz devices. In 2023, Qi *et al.* proposed a five-layer terahertz device architecture combining vanadium dioxide and graphene, which can switch between tunable circular dichroism (CD) and perfect absorption in two bands.^23^ Tang proposed an actively tunable and switchable multifunctional terahertz metamaterial device based on a hybrid vanadium oxide (VO_2_)-graphene integrated configuration. By switching the phase of vanadium dioxide, the device's functionality can be reversibly switched between asymmetric transmission (AT) and two different polarization conversions within the terahertz region.^24^ The phase change characteristics of vanadium dioxide are utilized to achieve the switchable performance between complete absorption and broadband asymmetric transmission (AT).^25^ Liu proposed a metamaterial doped with vanadium oxide (VO_2_) that can achieve switchable single-band and dual-band asymmetric transmission (AT) in the terahertz region.^26^ In the microwave range, Novoselov, one of the winners of the Nobel Prize for graphene discovery, has used printed graphene AMCs and flexible dielectric substrates to present absorbers with broadband effective absorption (more than 90% absorption) at 7.58 GHz to 18 GHz.^27^ The absorber is polarisation insensitive under normal incidence and can operate at relatively wide incidence angles. This amazing study is enough to prove that the research on THz devices is practically grounded and deserves more work to explore it in depth.

While a significant amount of related work has been reported, there exist challenges as well. For instance, it is difficult to achieve thermal radiation conditions when VO_2_ is placed in the middle layer.^28^ In addition to that, the change in temperature also affects the properties of graphene when VO_2_ and graphene are in the same plane.^29^

This paper innovatively presents a five-layer structure combining graphene and VO_2_, enabling a THz device to transition from narrowband to ultra-broadband absorption. The absorption can be tuned *via* voltage and temperature, and the unique structure ensures minimal interference between the dual-tuning effects of graphene and VO_2_. When VO_2_ is in its metallic state, the Fermi level of graphene is set to 0.8 eV, the THz device achieves perfect absorption with an absorptivity exceeding 90% across a bandwidth ranging from 2.9 to 7.67 THz. When VO_2_ is in its insulating state, the Fermi level of graphene is set to 1 eV, the THz device exhibits perfect absorption at 2.8 THz (*A* = 0.999) and 8.4 THz (*A* = 0.998). Furthermore, through our investigations, we discovered that when VO_2_ is in its insulating state, the operational performance of the THz device stems solely from the resonance generated by the graphene surface plasmons. When VO_2_ undergoes a phase transition to its metallic state, the absorption performance of the THz device is determined by the superposition of graphene and VO_2_. In summary, this THz device operating in narrowband mode can serve as a refractive index sensor and THz switch, while in broadband mode, it functions as an efficient THz absorber. This study provides novel research insights and references for subsequent investigations into multi-layer absorbers, as simulating and then combining graphene and VO_2_ separately can significantly reduce research time.

At present, a large number of related fields have been reported, but most of the research based on super surface terahertz devices is still in its initial stage. The manufacturability and practical application of the device need to be further discussed, and no systematic theory has been formed. The future research direction can combine machine learning to invert the process through the results. In addition, the precision of preparation process needs to be further considered to adapt to the actual application scenarios.

## 2. Structure design and unit model

**Fig. 1:**
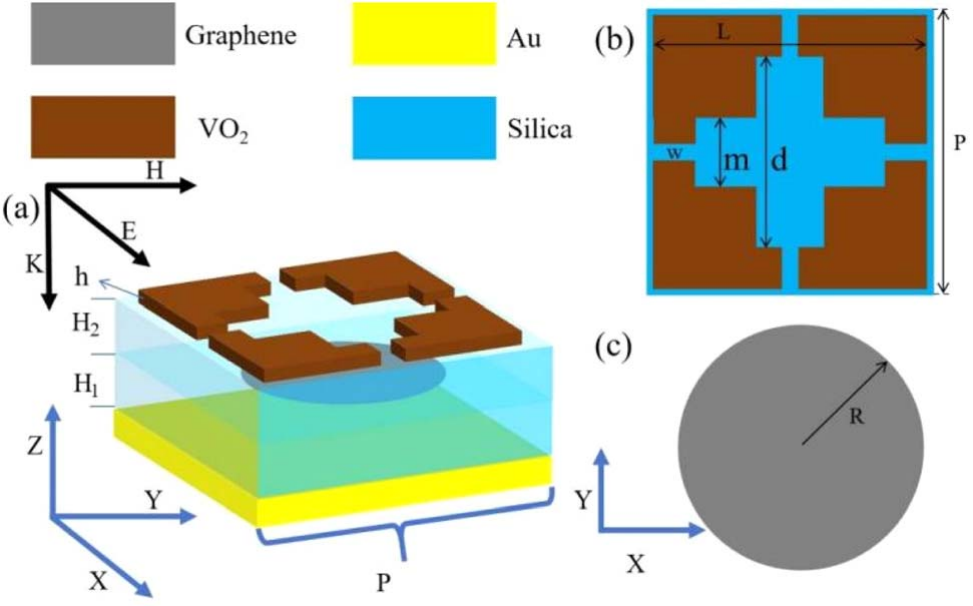
(a) Schematic of 3d model. (b) Schematic of VO_2_ layer. (c) Schematic of graphene layer.

The THz device proposed in this paper adopts a five-layer sandwich design, which consists of a VO_2_ film layer, a silica dioxide dielectric layer, a graphene layer, a silica dioxide dielectric layer and a gold layer. The three-dimensional schematic and geometrical parameters of the THz device are shown in Fig. 1(a)–(c) show the structure in plan view. The thickness of the bottom gold layer is 0.5 μm which is greater than the skin depth of gold. Gold is utilized as a reflective layer to ensure the confinement of electromagnetic waves within the device.^30^ Metal electrodes are connected to metal nanowires to modulate the Fermi energy levels of graphene layers with an external bias voltage.^31^ The thickness of SiO_2_ between the graphene layer and the VO_2_ film layer H2 = 4 μm, the dielectric layer is able to largely reduce the effect of by temperature on the properties of graphene, the dielectric constant of the two dielectric layers *ε* = 1.56. The top layer is VO_2_ thin film layer in centrosymmetric pattern with a thickness of 0.1 μm.

**Fig. 2:**
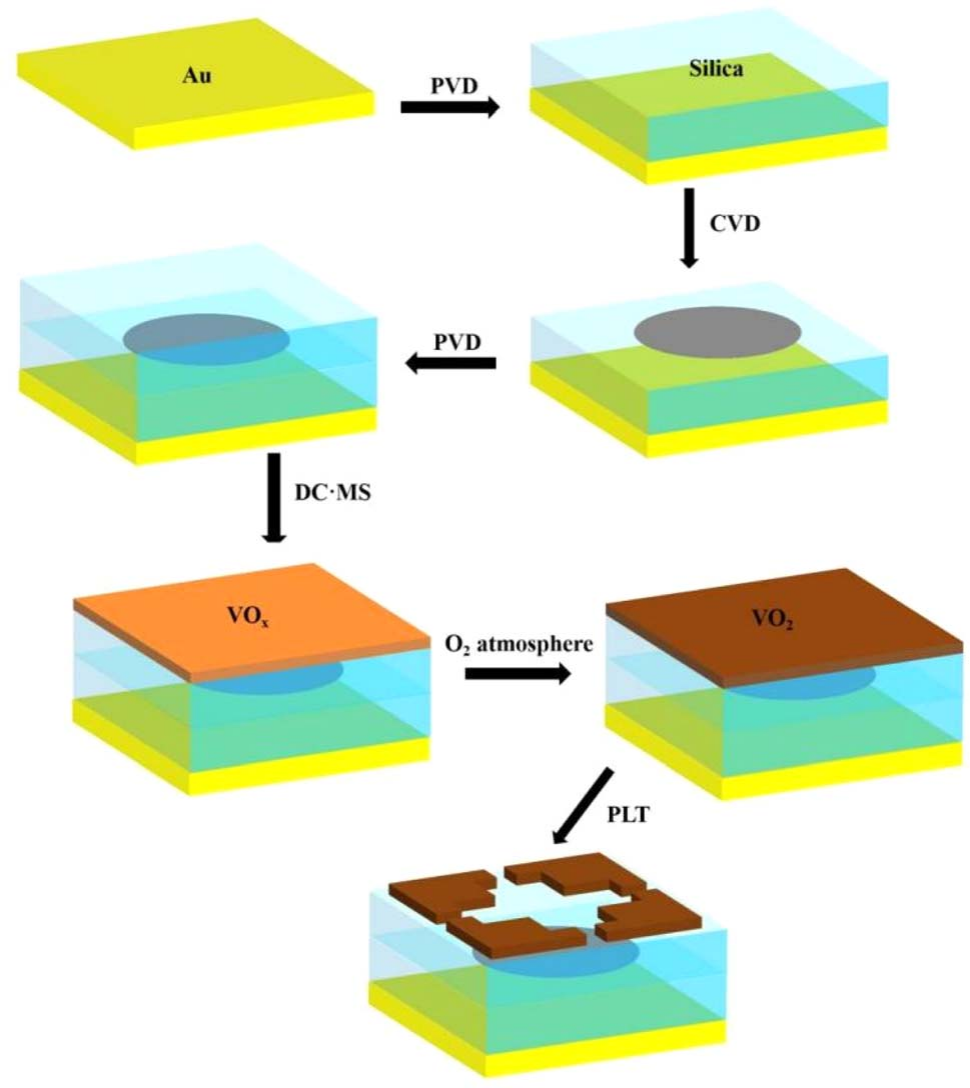
Preparation schematic and device preparation process.

The specific manufacturing process is shown with reference to Fig. 2. Firstly, the gold base (0.5 μm) is plated on the substrate. Next, a silica film with a thickness of 8 μm is deposited onto the gold substrate using physical vapour deposition (PVD) technology. Then, the desired graphene pattern was grown on the silica layer using CVD technique.^32^ The physical vapour deposition (PVD) technique was again used to coat the silica film with a thickness of 4 μm. Next, a 0.1 μm thick VO_*x*_ film was deposited on a glass film containing a vanadium(v) metal target using a direct current magnetron sputtering (DC, MS) method and further annealed in a low pressure O_2_ atmosphere to convert the VO_*x*_ to VO_2_.^29^ Finally, using a photolithography technique, a pre-bake and primer coating was performed before applying a uniform layer of photoresist on the VO_2_ film and pre-bake. The said VO_2_ film is aligned with the pattern mask described in the article and exposed. Finally, excess photoresist is removed, and device fabrication is complete.^33^

The specific structural parameters are shown in Table 1.

**Table 1:** Structural parameter diagram of the device

Parameter	*P*	*L*	*m*	*d*	*w*	*r*	*H*_1_	*H*_2_	*h*
Value (μm)	36	33	24	12	1.2	10	8	4	0.1

The conductivity of graphene can be classified into intraband and interband conductivity, which can be described as according to Kubo's formula:^34,35^

1 *σ*(*ω*, *μ*_c_, *Γ*, *Τ*) = *σ*_inter_ + *σ*_intra_

2
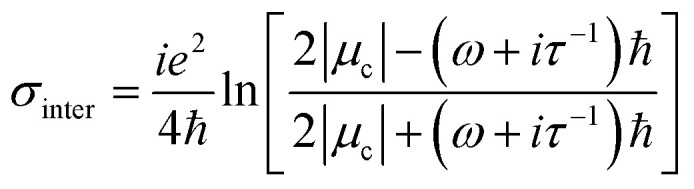
3
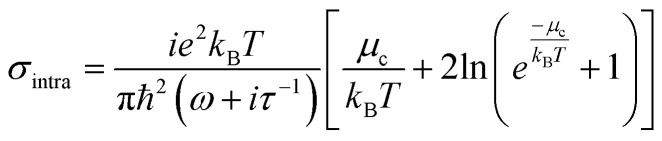
*ω*, *e* and *μ*_c_ are the angular frequency of terahertz electromagnetic waves, the electron charge, and the chemical potential of graphene. *τ* is the relaxation time, *T* is the Kelvin temperature, and *k*_B_ is the Boltzmann constant, and ℏ is the approximate Planck's constant. When *k*_B_*T* ≪ *μ*_c_, it can be approximated that when the graphene chemical potential is equal to the Fermi energy level, the interband conductivity can be simplified and expressed as:^36^4
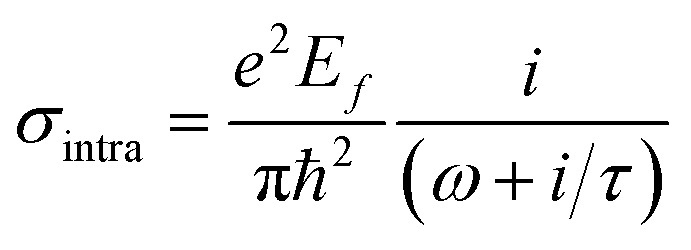


τ is the relaxation time of graphene, which is only relevant to the nature of the graphene material itself when the Fermi energy level of graphene is below the Dirac point.^37^ In this paper *τ* set to 0.4 ps, the graphene Fermi energy level can be adjusted by the applied bias voltage. *E*_*f*_ = 0.8 eV (*V*_g_ = 3.07 V) in broadband mode and *E*_*f*_ = 1 eV (*V*_g_ = 4.8 v) in narrowband mode.

In the terahertz range, we can express VO_2_ and gold in terms of the Drude model. The dielectric constant of gold can be expressed as:^38,39^5
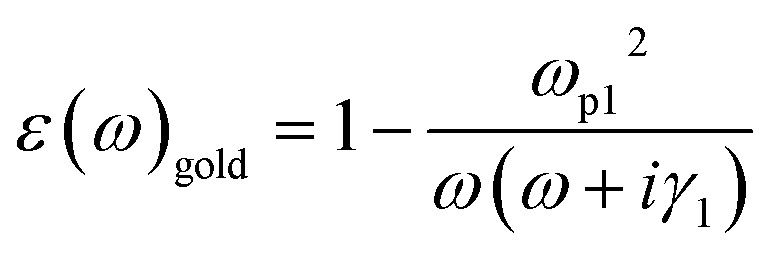
*ω*_p1_ = 1.37 × 10^16^ S^−1^, *γ*_1_ = 1.23 × 10^14^ S^−1^And for VO_2_, we can similarly use the Drude model to describe its dielectric constant in the terahertz range:^40^6
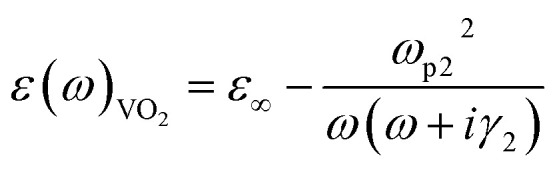
where *ω* is the frequency of the incident electromagnetic wave, and *ε*_∞_ = 12, denotes the relative permittivity of VO_2_ at infinity frequency. *γ*_2_ = 5.57 × 10^13^ is the collision frequency, where *ω*_p2_ is the plasma frequency associated with the conductivity of VO_2_, which can be approximated as:^41^7
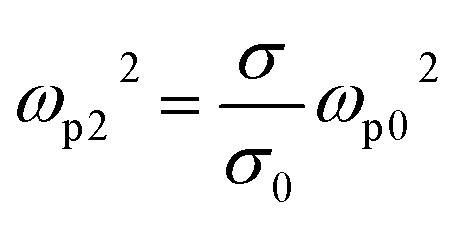
*σ*_0_ = 3 × 10^5^, *ω*_*p*0_^2^ = 1.45 × 10^5^. *σ* is the conductivity of VO_2_, when the temperature changes, VO_2_ will achieve the transition between the insulating state and the metallic state because of its unique phase transition property, and the relationship between the conductivity of VO_2_ and the ambient temperature is shown in Fig. 6(b). Before the phase transition, VO_2_ is in the insulating state with a conductivity of 2 × 10^2^ S m^−1^. When the temperature reaches the phase transition temperature, VO_2_ is in the metallic state with a conductivity of 2 × 10^5^ S m^−1^.

In this paper, the THz device model is simulated and verified using finite element analysis software CST Studio Suite.^42,43^ We construct the cell mesh in the *xy* coordinate plane, and the *z*-axis direction is the open boundary condition. The incident electromagnetic wave is incident from the positive half-axis of *z* to the negative half-axis of *z*. In addition to this, the simulation accuracy and precision are ensured by selecting the mesh refinement and turning on the mesh adaptive function.^44^

In CST Microwave Studio, the s-parameter of the model was calculated by simulation, and the absorption rate was expressed as:^45^

8 *A* = 1 − *R* − *T* = 1 − |*S*_11_|^2^ − |*S*_21_|^2^

*S*_11_ and *S*_21_ are the reflection and transmission coefficients, respectively, and *R* and *T* are the reflectance and absorbance. Since the bottom layer is a gold layer and the thickness of the gold layer is greater than the skin depth of gold, the transmittance can be considered to be 0 and the absorptivity formula can be simplified as:^46^

9 *A* = 1 − *R*

**Fig. 3:**
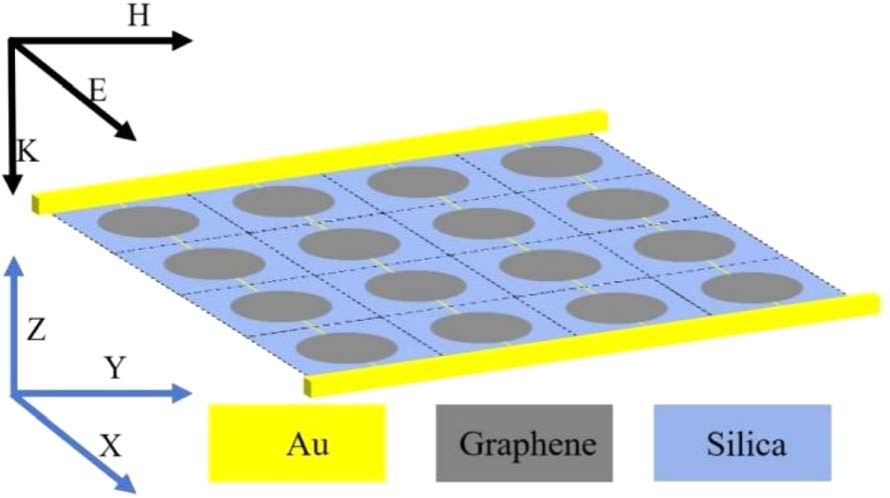
Schematic of graphene applied bias voltage.

The Fermi energy level of graphene is regulated by the applied bias voltage as shown in Fig. 3 and described in the article. In addition to this, the applied voltage to graphene is corresponding to the Fermi energy levels and the specific data can be presented by eqn (10).^47^10
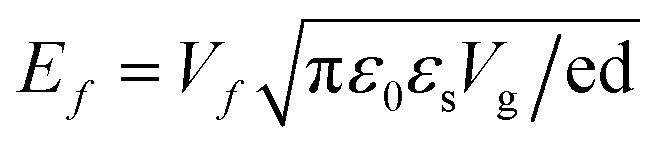


Fermi speed *V*_*f*_ = *c*/300.

## 3. Results and discussion

**Fig. 4:**
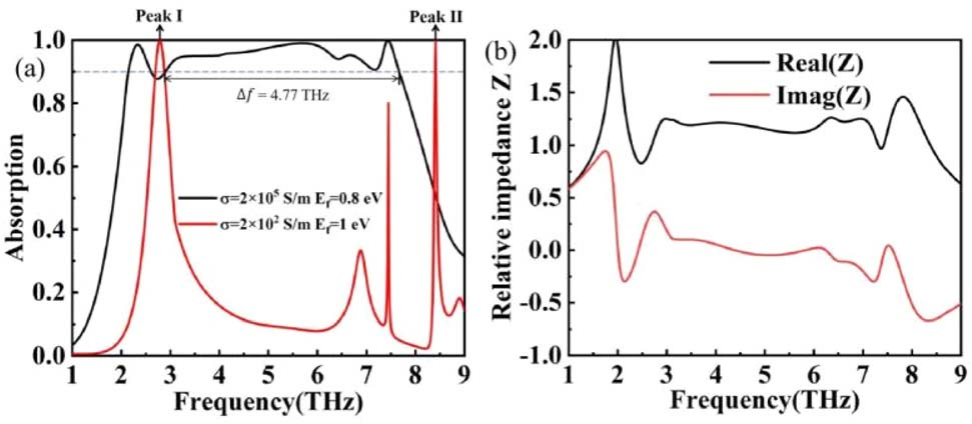
(a) Broadband and narrowband absorption curves, Peak I (*f* = 2.8 THz, *Q* = 5.28), Peak II (*f* = 8.41 THz, *Q* = 1020.5). (b) Relative impedance plot.

Through simulation work, we obtained the absorption spectra of this THz device in different modes, as shown in Fig. 4(a). The THz device has two absorption modes under the double tuning of VO_2_ phase transition properties and Fermi energy level. At room temperature (*T* = 300 K), VO_2_ is in the insulating state (*σ* = 200 S m^−1^) and the Fermi energy level *E*_*f*_ = 1 eV. The THz device has two absorption peaks, peak I (*f* = 2.8 THz) and peak II (*f* = 8.41 THz), and the absorption rates are 99.9% and 99.8% respectively, which can be approximately regarded as full absorption. In addition to this, the quality factor *Q* of peak I is 5.28 and that of peak II is 1020.5. When the temperature increases (*T* = 340 K), VO_2_ phase change (*σ* = 200 000 S m^−1^), Fermi level *E*_*f*_ = 0.8 eV, the absorber has an ultra wideband absorption mode, the absorber can achieve ultra wideband absorption in the range of 2.9–7.67 THz (*A* > 0.9). In the ultra-broadband absorption mode, in addition to the ultra-broadband absorption, there are two absorption peaks with an absorption rate of more than 95%.

To ensure the accuracy of the data, we introduce the relative impedance for inversion, which is expressed as:^48^11
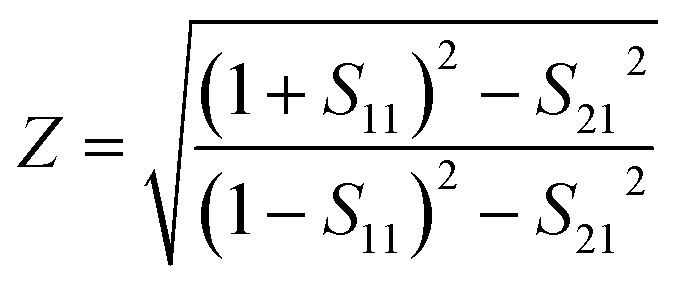
when the real part of the impedance Re (*Z*) = 1 and the imaginary part of the impedance Im (*Z*) = 0, the impedance of the THz device matches perfectly with the free-space impedance, and thus the perfect absorption is achieved, as shown in Fig. 4(b). It can be intuitively observed that the direction of the impedance curve corroborates with higher absorption regions in the absorption map. Specifically, within the broadband absorption range, the real part of the impedance tends to 1, while the imaginary part tends to 0.

**Fig. 5:**
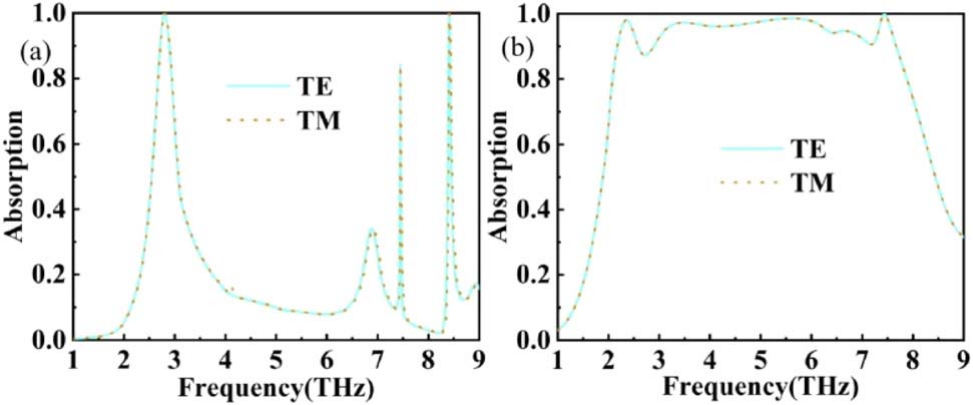
(a) Absorption curves of vertically polarised (TE-wave) and parallel polarised (TM-wave) electromagnetic waves at vertical incidence *σ*_VO_2__ = 2 × 10^2^ S m^−1^, *E*_*f*_ = 1 eV. (b) Absorption curves of vertically polarised (TE wave) and parallel polarised (TM wave) electromagnetic waves at vertical incidence *σ*_VO_2__ = 2 × 10^5^ S m^−1^, *E*_*f*_ = 0.8 eV.

Meanwhile, we also verified the change of absorbance in two polarisation modes (TE and TM), as shown in Fig. 5(a) and (b), and since the structure proposed in this paper is a fully symmetric structure, it can maintain the same excellent absorption performance in either TE or TM modes, which also indicates the great potential of this THz device for application.^49,50^ Due to this characteristic, the subsequent discussions in this paper will primarily focus on the TE mode.

The strong tuning ability of this THz device is due to the phase transition property of VO_2_. In the CST software, we can change the temperature by setting the conductivity of VO_2_ at different temperatures, which can be described by the formula:^51^

**Fig. 6:**
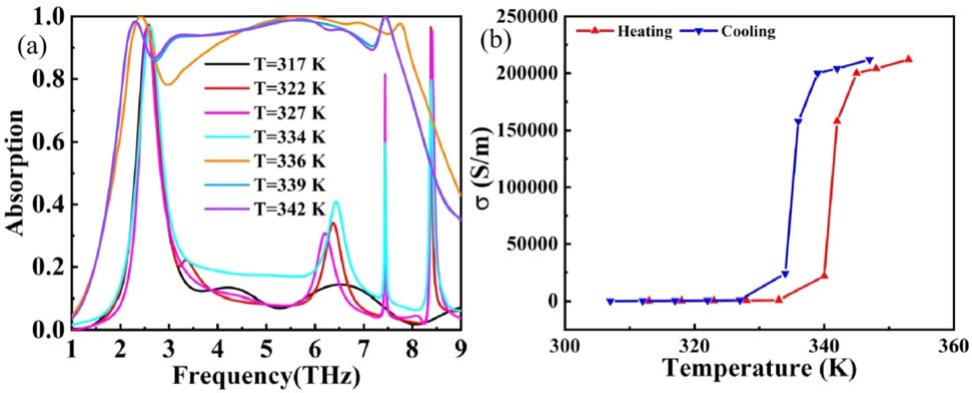
(a) Absorption curves corresponding to different temperatures when the THz device is heated. (b) Conductivity *versus* temperature curves for VO_2_ films.

12 *σ* = −*iε*_0_*ω*(*ε*_c_ − 1)

where *ε*_0_ and *σ* are the vacuum dielectric constant and the conductivity of VO_2_, and *ε*_c_ is the temperature-dependent dielectric function. In practical applications, the temperature can be regulated by inlaying resistance wires around the device package. Since the phase transition temperature is only 68 °C, the heat generated by the resistor wire can reach the temperature requirement of the phase transition. During the insulator-metal phase transition, the conductivity of VO_2_ increases steadily and can be varied in the range of 2 × 10^2^ S m^−1^ to 2 × 10^5^ S m^−1^, while the crystal structure of VO_2_ changes from a monoclinic to an orthorhombic structure, as shown in Fig. 6(b). In summary, a change in temperature allows for a VO_2_ insulating to metallic phase change process to achieve different functions.

The variation of vanadium dioxide's conductivity with temperature is illustrated in Fig. 6(b), where the red line represents the change in conductivity during heating, and the blue line represents the change during cooling. When *T* = 340 K (approximately 68 °C), VO_2_ undergoes a phase transition from an insulating state to a metallic state. Concurrently, the absorption mode of the THz device transitions from narrowband perfect absorption to broadband absorption, as shown in Fig. 6(a).

**Fig. 7:**
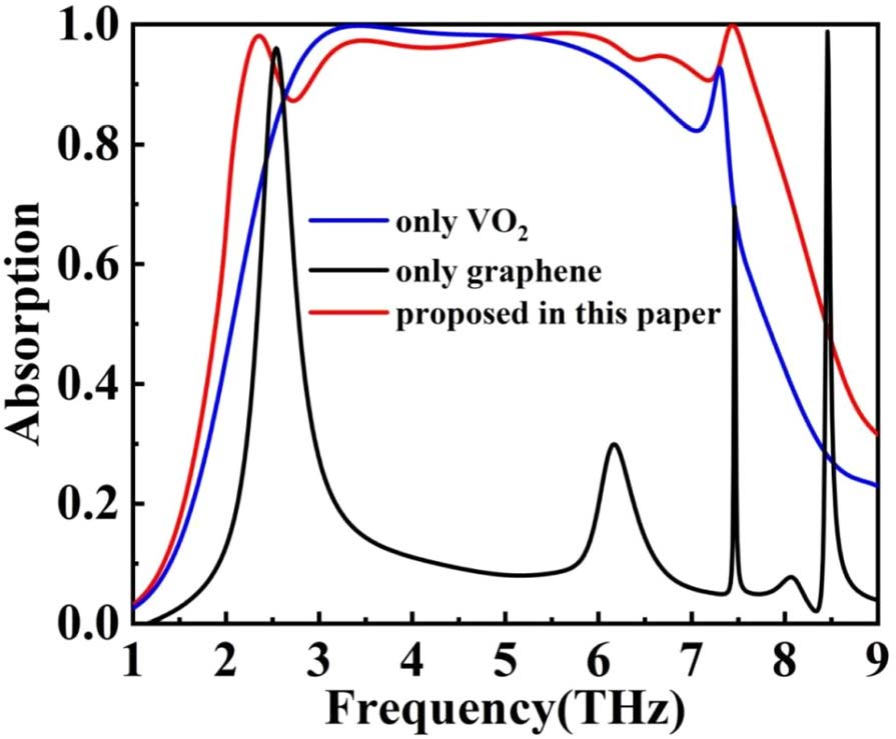
Absorption rates for different terahertz device configurations.

To investigate the relationship between the structure and absorption of this THz device, we employed the control variable method to study the interplay between VO_2_ and graphene, as depicted in Fig. 7. By replacing the graphene disk with a silica medium, the VO_2_ structure alone was able to achieve broadband absorption with a bandwidth of approximately 3 THz and an absorptivity greater than 0.9. In contrast, when the THz device contained only the graphene structure, it exhibited two distinct absorption peaks.

The absorption curve of the THz device with only VO_2_ pattern changes similar to that of the THz device proposed in this paper (after phase transition). The absorption curve of the THz device with only a graphene pattern changes similarly to that of the THz device proposed in this paper (before phase transition). When we combine the two, we can get a perfect ultra-broadband THz device (after phase transition) along with a double-peaked narrow-band perfect absorption (before phase transition). From this, it can be inferred that the interaction of graphene structure and VO_2_ structure with electromagnetic waves can be approximated as working independently and that in the narrow-band absorption mode (before VO_2_ phase transition), it is mainly the interaction of graphene with electromagnetic waves, and in the ultra-broadband absorption mode (after VO_2_ phase transition) it can be considered to be the superposition of the interaction absorption of the two materials.^52,53^

**Fig. 8:**
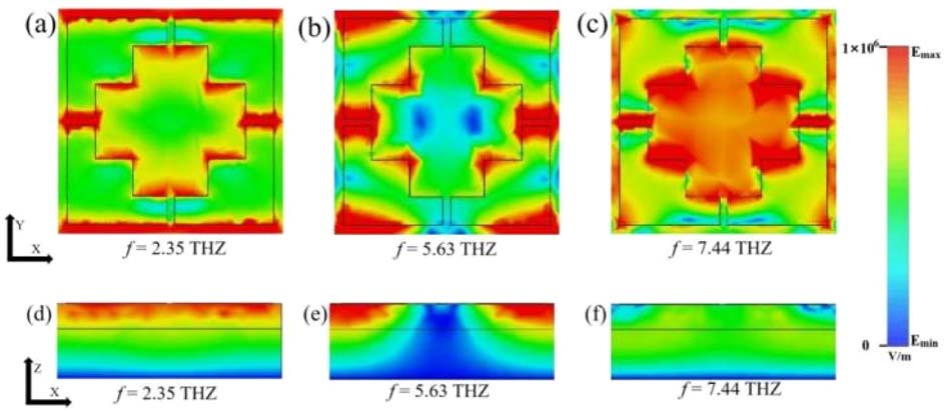
(a)–(c) show the electric field of the terahertz device in the *xy*-direction cross-section at frequencies of 2.35 THz, 5.33 THz and 7.41 THz, respectively. (d)–(f) show the electric field in the *xz*-direction cross-section of the terahertz device for frequencies of 2.35 THz, 5.33 THz and 7.41 THz, respectively.

In order to further investigate the mechanism of action of the THz device, it is explored by studying its electric field diagrams, as shown in Fig. 8(a)–(f). Fig. 8(a)–(c) show the electric field strength absorption distribution of the xy-plane of the THz device at 2.35 THz, 5.63 THz, and 7.44 THz, and Fig. 8(e)–(f) show the electric field strength absorption distribution of the *xz*-plane of the THz device at 2.35 THz, 5.63 THz, and 7.44 THz. The strong absorption of terahertz waves by the THz device originates from the electromagnetic wave interacting with the THz device structure and coupling with the structure pattern to form localized surface plasmon resonance (LSPR).^54,55^

By comparing and analyzing Fig. 8(a)–(c), (e) and (f), in the low-frequency absorption band (*f* = 2.35 THz), the electric field primarily concentrates at the edges of the VO_2_ module and within the slits, accompanied by electric dipole resonance absorption at the edges of the graphene disk. This leads to the dissipation of incident light energy within the upper layer structure, enabling the formation of perfect absorption in the THz device.^56^ As the frequency increases (*f* = 5.63 THz), the intense absorption electric field is primarily attributed to the coupling between VO_2_ modules, accompanied by electric dipole resonance absorption at the edge portions of the graphene disk. The electric field primarily concentrates within the periodic structures.^57^ During the high-frequency band absorption (*f* = 7.44 THz), the intense absorption electric field shifts inward, reducing the influence of the edges of the VO_2_ modules and subsequently diminishing the absorption performance. At this point, the role of the graphene structure becomes particularly prominent, as the electric dipoles at the edges of the graphene disk resonate with the high-frequency band, resulting in high absorption.^58^ By comparing and analyzing Fig. 7 and 8, it is easy to find that the high absorption in the narrow-band mode is almost always caused by the graphene disc, while the ultra-wideband mode is more like a superposition state of the VO_2_ structure and the graphene structure.

**Fig. 9:**
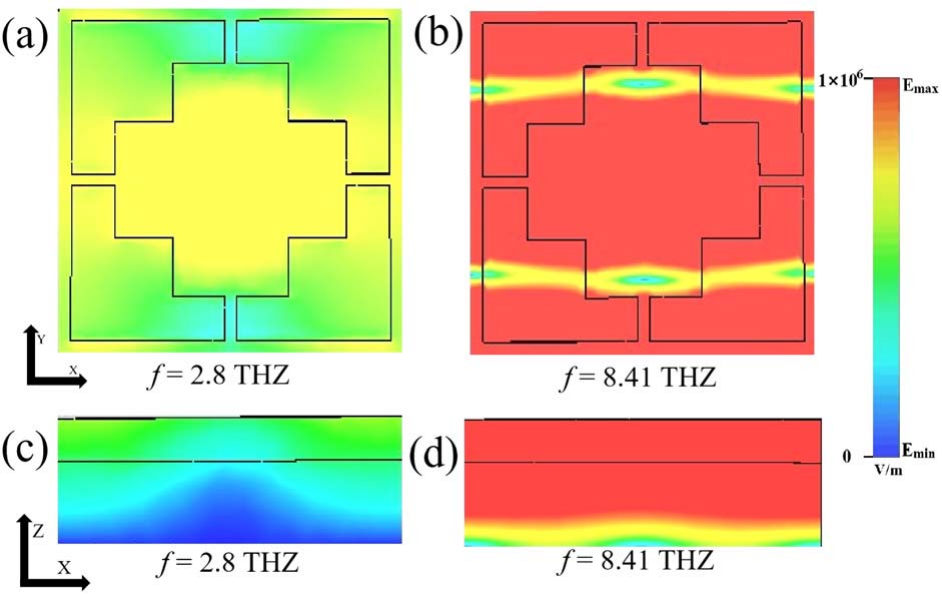
(a) and (b) show the electric field in the *xy*-direction cross-section of a terahertz device at frequencies of 2.8 THz and 8.41 THz. (c) and (d) show the electric field in the *xz*-direction cross-section of a terahertz device at frequencies of 2.8 THz and 8.41 THz.

Fig. 9(a) and (b) show the electric field distribution of the *xy* cross section of the device at 2.8 THz and 8.41 THz frequencies, and (c) and (d) show the electric field distribution of the *xy*-direction cross-section of the device at 2.8 THz and 8.41 THz frequencies. By comparing the *xy*-plane and *xz*-plane absorption electric field maps, we can get a very obvious conclusion. The absorption in the low-frequency band is primarily concentrated within the graphene structure, whereas the absorption performance of the graphene structure is less ideal in the high-frequency band. In the narrowband absorption mode, VO_2_ is in an insulating state. Under such conditions, the absorption in the low-frequency band (*f* = 2.8 THz) arises from edge dipole resonance, while the absorption in the high-frequency band (*f* = 8.41 THz) stems from the resonant absorption of the graphene disk. Additionally, anti-parallel currents between the graphene sheet and the underlying metal layer are forming a loop, generating an artificial magnetic moment, leading to the emergence of other peaks.^59^

**Fig. 10:**
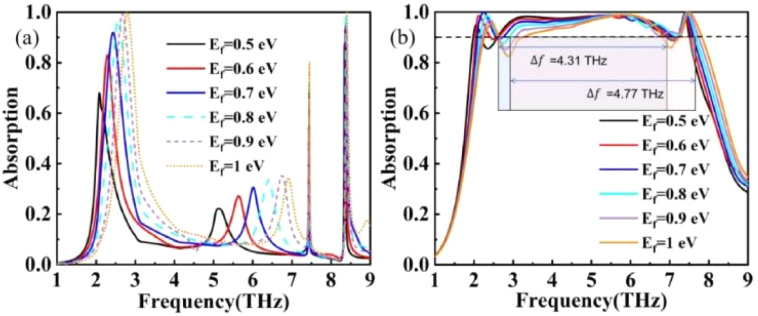
(a) Effect of Fermi energy levels on absorption rate in narrowband absorption mode. (b) Effect of Fermi energy levels on absorption rate in broadband absorption mode.

As depicted in Fig. 10, the wide tuning capability of the absorber also originates from the tuning of the Fermi level in graphene. By adjusting the Fermi level, it is possible to achieve tuning of narrowband absorption and expansion of broadband absorption. As can be observed from Fig. 10(a), the absorption of the two absorption peaks can be adjusted by tuning the Fermi level. When the Fermi level is at a lower energy level, absorption peak I exhibits a lower absorption level. As the Fermi level gradually increases, the absorption rate gradually rises, reaching a maximum of 0.9 at *E*_*f*_ = 0.7 eV. When the absorption is at a reduced level, the THz device can be considered closed, and when the absorption is greater than 0.9, the THz device can be considered open. In addition to the switching characteristics of the Fermi energy level tuning in the narrow-band mode, the Fermi energy level tuning can also be used for tuning in the broadband mode. As shown in Fig. 10(b), the THz device absorption width is only 4.31 THz when *E*_*f*_ = 0.5 eV, while the THz device broadband absorption expands to ultra-wideband absorption (Δ*f* = 4.77 THz) when the Fermi energy level grows to 0.8 eV.

**Fig. 11:**
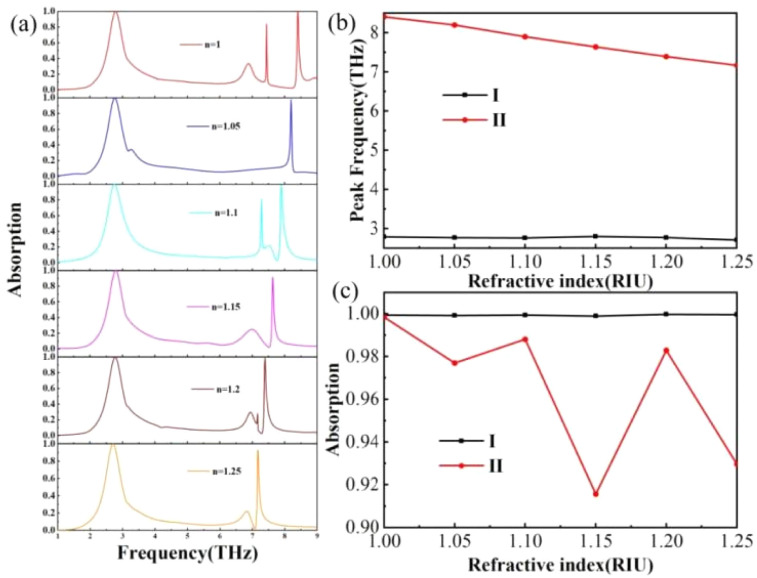
(a) Change in absorption spectrum of the terahertz device when the ambient refractive index increases from 1 to 1.25. (b) Change in the frequency of the absorption peak when the ambient refractive index is increased from 1 to 1.25. (c) Change in absorbance of the absorption peak when the ambient refractive index is increased from 1 to 1.25.

Furthermore, due to the high-quality factors of the two absorption peaks of THz devices under narrowband mode, they hold promising potential applications in sensing and detection. Specifically, THz devices can be utilized as ultra-sensitive atmospheric refractive index sensors.^60^Fig. 11 illustrates the absorption behavior of the THz device under varying environmental refractive indices. As the environmental refractive index gradually increases, a red shift occurs in peak II, accompanied by changes in its absorption rate. However, the position of peak I remains largely unchanged. This could be attributed to the fact that the absorption of peak I is primarily determined by the graphene structural layer, which is in a closed state and cannot be in contact with the external environment. Consequently, variations in the refractive index of the natural external environment do not affect the absorption of peak I. The absorption rate of peak II varies with the atmospheric refractive index, and its absorption frequency exhibits a linear relationship with the refractive index of the environment. Based on this discovery, we can monitor changes in the atmospheric refractive index by observing shifts in the resonant frequency of the absorption peak, thereby achieving effective atmospheric refractive index detection. We can measure the performance of this terahertz device based on sensitivity (S) and quality, and the metric can be described as:^61^13
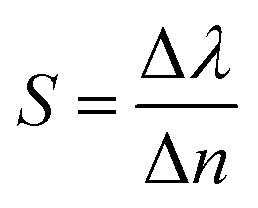
where Δλ is the amount of change in the resonance wavelength of the absorption peak relative to the change in the ambient refractive index. Δ*n* is the amount of change in the ambient refractive index. According to the calculation, the sensitivity of peak II can be calculated as 4958 GHz/RIU, the absorption peak is very sensitive to the change of the ambient refractive index, which indicates that the terahertz device has a great potential for application in refractive index sensors.^62^

**Fig. 12:**
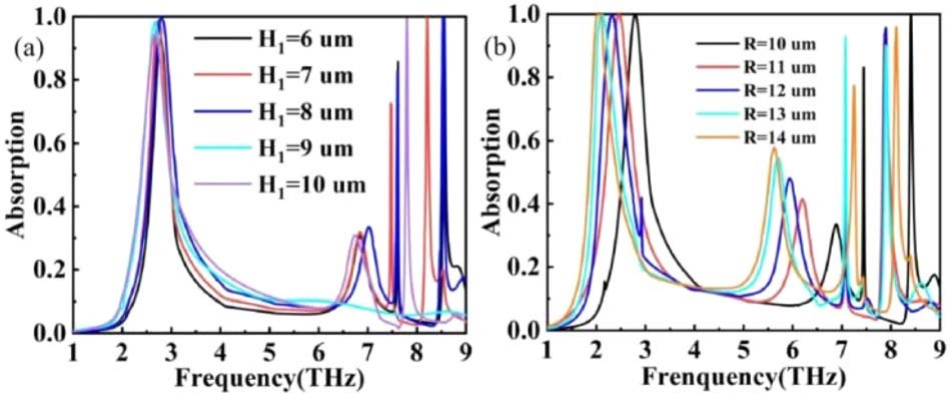
(a) Effect of silica dielectric layer H_1_ on absorption rate. (b) Effect of graphene disc radius *R* on absorption rate.

In addition to studying the applications and functionalities of terahertz devices, the structural parameters of these devices also constitute a critical component of terahertz research, particularly in relation to their parameter insensitivity characteristics. Manufacturing errors are inevitable during the fabrication process of terahertz devices. By simulating the structural parameters of terahertz devices, we can observe changes in their absorption curves. Varying the height of the dielectric layer, reveals that no absorption peak is generated when H_1_ = 9 μm, while the remaining parameters exhibit good absorption, as shown in Fig. 12(a). Altering the radius of the graphene disk results in a red shift of the absorption curve and a decrease in absorption rate. This phenomenon is attributed to the fact that the electric dipole resonance frequency at the edges of the graphene changes with the increase in *R*.

**Fig. 13:**
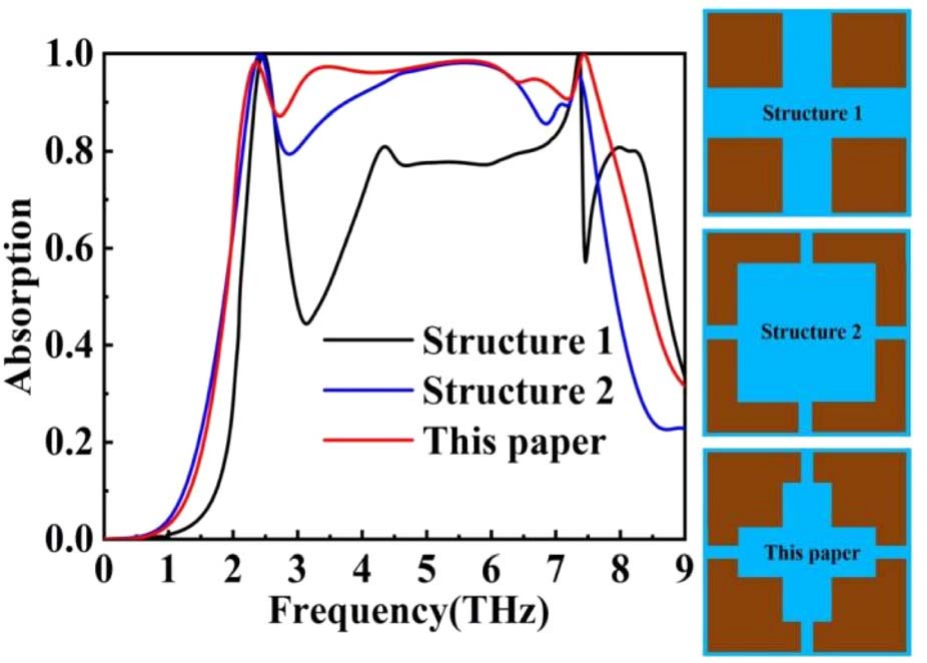
Absorption curves for different structures.

The design of this device is based on the characteristic dimensions of current manufacturing technology, and its structural parameters represent the optimal solution derived from simulation results. This absorber can only produce ultra-broadband absorption with a specific structure, and its superior performance is attributed to resonant absorption. By altering the structure, we can obtain outcomes worthy of further research. As illustrated in Fig. 13, neither Structure 1 nor Structure 2 achieves satisfactory absorption.

**Fig. 14:**
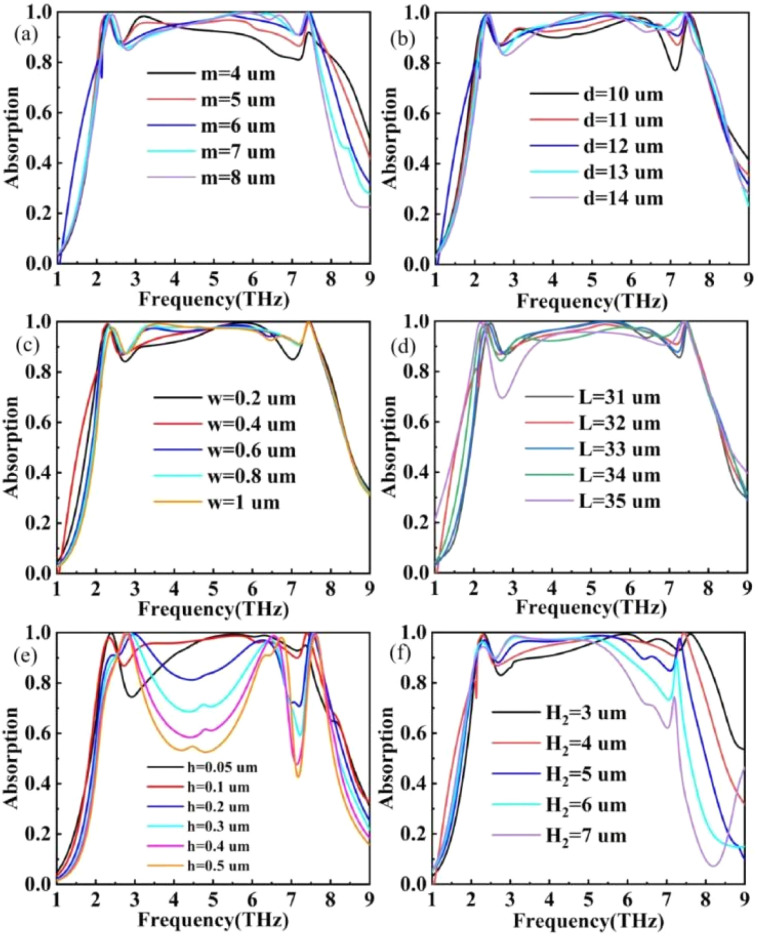
(a)–(e) Effect of VO_2_ pattern parameters *m*, *n*, *w*, *L*, and *h* on absorption rate, respectively. (f) Effect of dielectric layer silica thickness H_2_ on absorption rate.

Fig. 14(a)–(e) show the variation of terahertz device absorption curves when the structural parameters of VO_2_ are varied. As the *m* parameter increases, the high-frequency portion of the absorption band undergoes a redshift, accompanied by an enhancement in resonance intensity. Similar variations are observed in the *d* parameter, as depicted in Fig. 14(a) and (b). As the width (*w*) of the slit increases, the plasmonic resonance between VO_2_ modules intensifies, resulting in high absorption, as illustrated in Fig. 14(c). When the *L* parameter varies, the absorption curves are shown in Fig. 14(d). When *L* is 35 μm, the length of the VO_2_ modules becomes similar to that of the overall structure, leading to a decrease in electric dipoles at the edges and a subsequent reduction in resonant absorption. The influence of VO_2_ thickness was investigated, and the optimal resonant absorption was observed only when *h* = 0.1 μm. As the thickness of the dielectric layer (H_2_) increases, the high-frequency portion of the absorption band undergoes a redshift, resulting in a decrease in absorption efficiency.

In the research of terahertz devices, it is also crucial to investigate the influence of electromagnetic wave incident angle and polarization angle.^63–66^ To investigate the absorption response of terahertz devices under oblique incidence conditions, the variations in incident angle were studied separately in both broadband and narrowband modes. In the simulation, the incident angle refers to the angle between the incident electromagnetic wave and the *Z*-direction, ranging from 0° to 70°. When the incident angle is less than 50°, the broadband absorption mode maintains excellent absorption characteristics, while a blue shift occurs when the incident angle exceeds 50°. When the incident angle is less than 60° degrees, the narrowband absorption mode maintains good absorption characteristics. However, when the incident angle exceeds 60°, the absorption effect decreases.

**Fig. 15:**
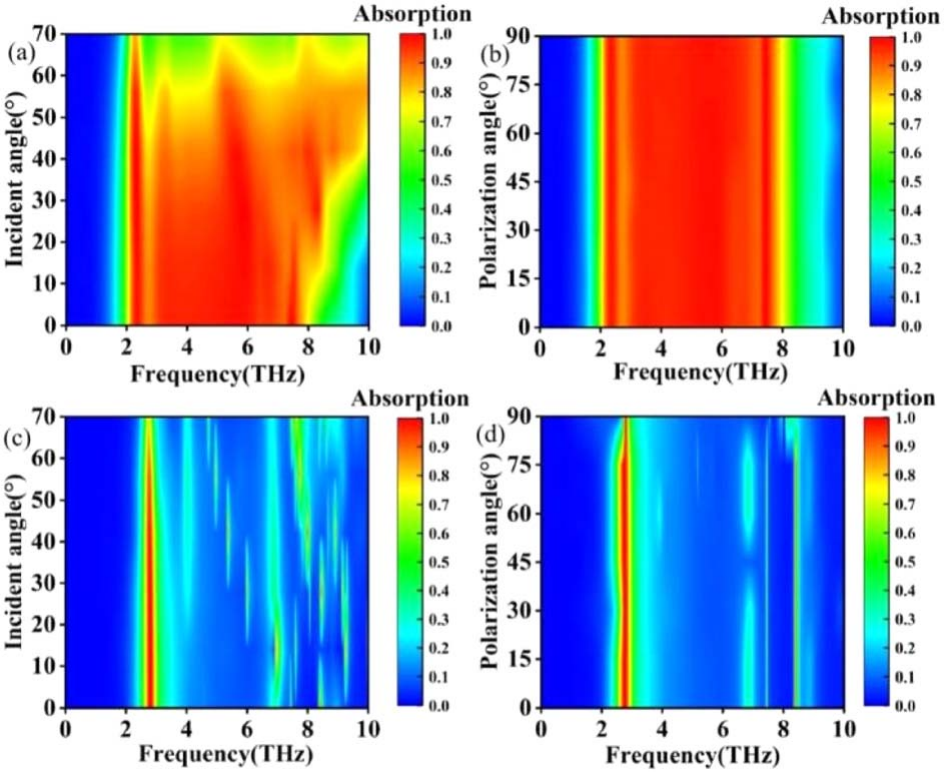
(a) Absorption spectra at different incidence angles in broadband absorption mode. (b) Absorption spectra in broadband absorption mode for different polarisation angles. (c) Absorption spectra for different incidence angles in narrowband absorption mode. (d) Absorption spectra in narrowband absorption mode at different polarisation angles.

When the polarisation angle of the incident wave is varied in the range of 0° to 90°, the absorption spectrum of the terahertz device remains basically unchanged, as shown in Fig. 15(b) and (d). In order to study the change in absorbance under the change in polarisation angle, the range of polarisation angle was set to 0°–90° with a gradient of 10°. In broadband absorption mode, the absorption spectrogram is basically unchanged when the polarisation angle is varied in the range of 0°–90°, as shown in Fig. 15(b). Under the narrowband absorption mode, the absorption spectrum remains largely unchanged as the polarization angle varies within the range of 0°–75°. However, the absorption bandwidth narrows when the angle exceeds 75°, as shown in Fig. 15(d). As can be seen from the above, the terahertz device proposed in this paper is insensitive to the polarization angle.^67,68^

In recent years, VO_2_ and graphene have been widely used in terahertz absorbers. Multifunctional devices such as narrow-band absorption, broadband absorption, and switching between narrow-band absorption and broadband absorption achieved by terahertz absorbers based on these two materials have been reported extensively. Compared to the various devices listed in Table 2, our devices have better performance in broadband absorption as well as narrowband absorption.^69–73^ Overall, the devices proposed in this paper have wider and superior performance.

**Table 2:** Comparisons between the proposed structure and other publications

Reference	Materal	Layers	Functions (absorption band)	Broadband bandwidth (THz)	Absorptance (%)
69	VO_2_	4	Multiband (three peaks) & broadband	0.398–1.356	>90
70	Dielectric & VO_2_	3	Broadband	1.2–6.1	>90
71	Graphene & VO_2_	7	Multiband (three peaks) & broadband	0.8–2.4	>90
72	Graphene & VO_2_	3	Broadband	3.58–8.2	>90
73	Graphene & VO_2_	5	Multiband (two peaks) & broadband	3.26–6.91	>90
This work	Graphene & VO_2_	5	Multiband (two peaks) & broadband	2.9–7.67	>90

## 4. Conclusion

In summary, a multifunctional terahertz device based on a hybrid structure of VO_2_ and graphene is proposed in this paper. The terahertz device can achieve various functions by utilizing the phase transition characteristics of VO_2_ and the adjustment of the Fermi level of graphene. When VO_2_ is in its metallic state and the Fermi level of graphene is 0.8 eV, the device can function as an absorber with a bandwidth ranging from 2.9 to 7.67 THz (*A* >0.9). Moreover, this terahertz device exhibits a wide range of incident angle insensitivity, maintaining good absorption performance when the incident angle of the electromagnetic wave is less than 50°. Adjusting the graphene Fermi energy level from 0.8 eV to 1 eV when VO_2_ is in the insulating state enables perfect narrow-band absorption. The dual-peak under narrowband absorption can be utilized as a sensing switch and refractive index sensor detector. The terahertz device in narrow-band mode has a strong polarisation insensitivity and operates well over a wide range of incidence angles. Overall, the terahertz device is able to achieve multiple functional device applications through dual tuning of temperature and bias voltage, and the terahertz device has great application prospects in terahertz wave absorption, environmental monitoring, and optical light opening.

## Conflicts of interest

The authors declare that they have no known competing financial interests or personal relationships that could have appeared to influence the work reported in this paper.

## Data Availability

The data are available from the corresponding author on reasonable request.
